# Concurrent Tuberculous Meningoencephalitis and Anti-NMDAR Encephalitis: A Case Report

**DOI:** 10.3389/fneur.2022.870607

**Published:** 2022-07-07

**Authors:** Chen Xiaoli, Wang Qun, Li Jing, Yang Huan, Chen Si

**Affiliations:** ^1^Department of Neurology, Shaanxi Provincial People's Hospital, Xi'an, China; ^2^Department of Neurology, Xiangya Hospital, Central South University, Changsha, China; ^3^National Clinical Research Center for Geriatric Disorders, Xiangya Hospital, Central South University, Changsha, China

**Keywords:** anti-NMDAR antibody, autoimmune encephalitis, case report, *Mycobacterium tuberculosis*, tuberculosis meningoencephalitis

## Abstract

**Background:**

Cases of tuberculosis triggering the development of anti-*N*-methyl-*D*-aspartate receptor (NMDAR) encephalitis are absent.

**Case Presentation:**

Herein, we report, for the first time, the case of a patient who developed anti-NMDAR encephalitis likely due to tuberculosis. The patient, a 33-year-old man, experienced weight loss during the previous 2 years, along with acute headache, fever, cognitive deficits, and right ophthalmoplegia. Based on these findings and on data from magnetic resonance imaging and cerebrospinal fluid antibody analysis, tuberculous meningoencephalitis combined with anti-NMDAR encephalitis was diagnosed. Marked clinical and brain imaging improvement were observed after antituberculosis and high-dose corticosteroid treatment initiation, which persisted during the 3 months of follow-up.

**Conclusions:**

This case suggests that anti-NMDAR encephalitis may arise after tuberculosis infection. Therefore, clinicians must be aware of this possibility, especially when cognitive and new neurological symptoms suddenly occur.

## Introduction

Encephalitis caused by antibodies against the *N*-methyl-*D*-aspartate receptor (NMDAR) is a well-described dysimmune entity of the central nervous system (CNS) and the most frequent form of autoimmune encephalitis (AIE) (1). Frequently idiopathic, anti-NMDAR encephalitis can occur as a paraneoplastic manifestation and post-infection neurological complication (2). In particular, the herpes simplex virus (HSV) is the most frequently identified virus associated with this disorder (3). This study describes the case of a patient presenting with anti-NMDAR encephalitis after a *Mycobacterium tuberculosis* (MTB) infection.

### Case Presentation

**Figure 1:**
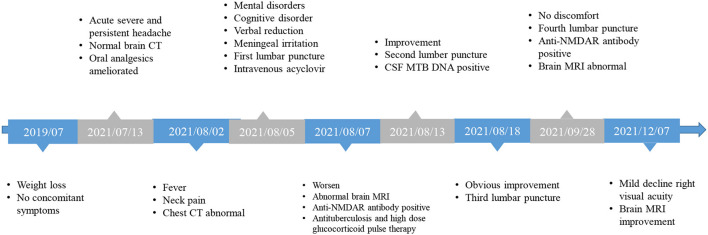
Timeline of the clinical course.

**Figure 2:**
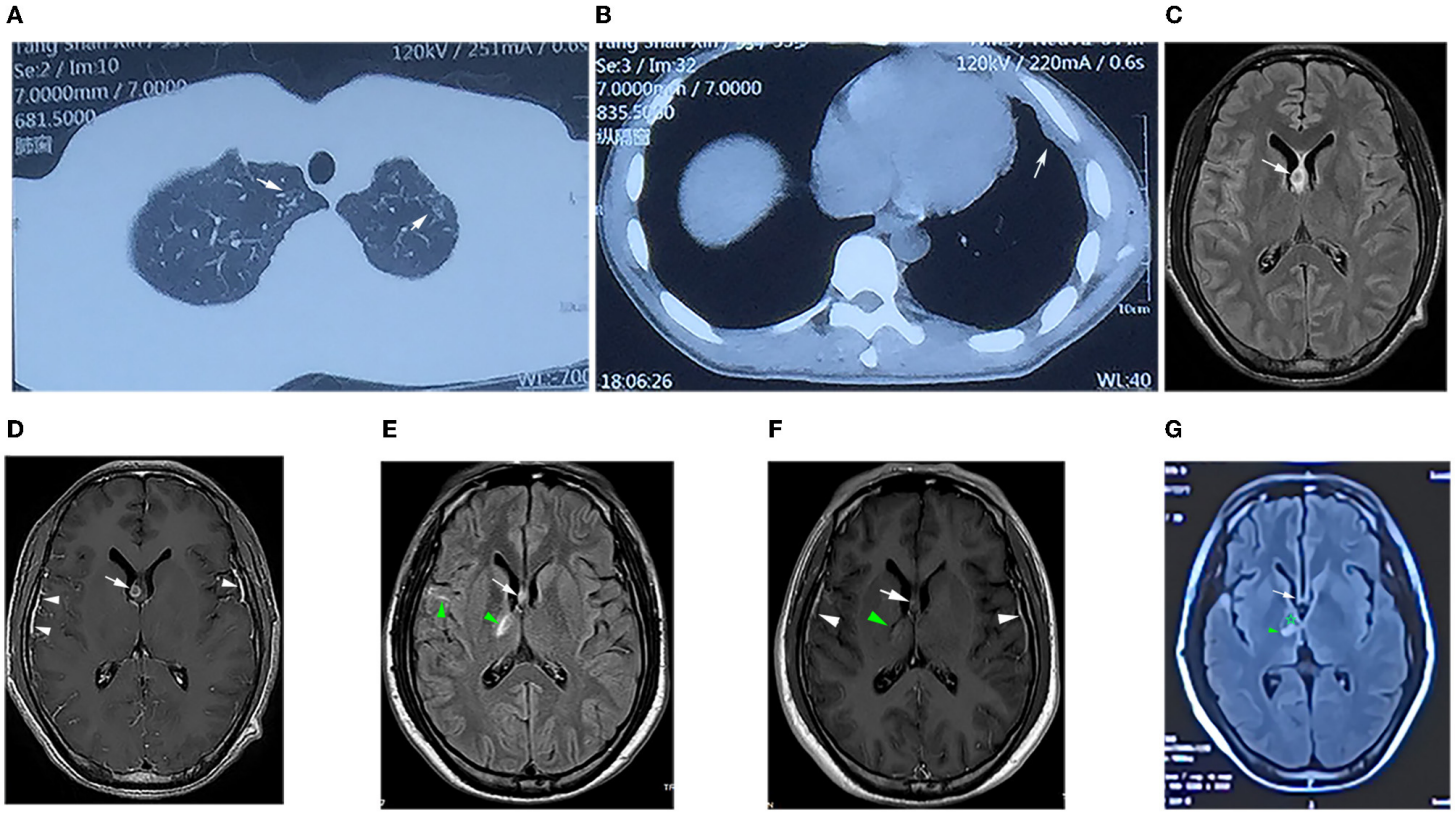
**(A)** Computed tomography (CT) of the chest showing patchy high-density shadows in the upper lobes of both lungs (white arrow), suggesting secondary to tuberculosis. **(B)** CT scan revealed thickening of the left pleura and encapsulated effusion (white arrow). **(C)** T2-weighted magnetic resonance imaging (T2WI) FLAIR showing small circular isointense, slightly long hyperintense in the right lateral ventricle subependymal (white arrows). **(D)** Contrast-enhanced T1WI showing that the lesion was ring-enhancing (white arrows) and significant enhancement of the bilateral temporal leptomeningeal (white triangle). **(E)** T2WI FLAIR image showing that the right subependymal lesion (arrow) was smaller than that in **(C)**, and the right basal ganglia and right temporal lobe were patchy and line-like hyperintensity (green triangles). **(F)** Contrast-enhanced T1WI showing that the lesion in the right subependymal was smaller than that in **(D)** (white arrow), the lesion in the right basal ganglia was slightly enhanced (green triangle), and the meninges were thicker as before (white triangle). **(G)** T2WI FLAIR image showing that the subependymal lesion had basically disappeared (white arrow), the right basal ganglia lesion was slightly smaller (green triangle), and softening could be seen inside (✰).

The patient, a 33-year-old man, was hospitalized with complaints of headache, fever, unresponsiveness, irritability, and neck pain. The patient was reported to have lost 35 kg during the last 2 years (Figure 1). Twenty days before admission, the patient experienced persistent acute headaches, primarily in the front of the ear, temporal and posterior occipital part of the right side, without any distinct cause. These headaches were mainly characterized by unbearable distending pain, occasionally accompanied by dull, tingling, throbbing pain, which led to prolonged insomnia. Moreover, the patient experienced loss of appetite, but without nausea, vomiting, fever, diplopia, limb movement disorder, chest tightness, or shortness of breath. Eighteen days before admission, the patient sought medical help to manage the headaches. At that time, brain computed tomography (CT) scans were normal and the patient was given analgesic drugs, including rotundine (60 mg, orally three times per day), which relieved the headaches for 4 h and partly ameliorated the insomnia. Gradually, the degree of the headaches reduced, only occurring three to four times a day for approximately 4 h. However, 3 days before admission, the patient developed a low-grade fever when a headache occurred, accompanied by irritability and neck pain. Brain MRI was normal, but chest CT images revealed that the upper lobe of both lungs had lesions secondary to tuberculosis, and the left pleura was thickened and had encapsulated effusion (Figures 2A,B). The patient had no history of chronic disease, smoked for 20 years (10 cigarettes per day), occasionally drank a small amount of alcohol, and denied to have a family history of genetic disease. At admission, the patient was irritable and unresponsive, and upon examination, verbal reduction (unable to express full sentences), mild loss of comprehension, decreased calculation ability, and poor spatial learning and memory were confirmed. The eyeballs were in place on both sides, and horizontal nystagmus to the right was observed. The patient also showed signs of meningeal irritation, with nuchal rigidity (neck flexion angle of approximately 5°), positive bilateral Kernig's sign, and negative Brucella's sign. According to the Clinical Assessment Scale in AIE (CASE) (4), modified Rankin scale (mRS), Glasgow coma scale, mini-mental state examination (MMSE), and the Montreal Cognitive Assessment Test (MoCA) the patient scored 6, 3, 13 (E3M4V6), 9, and 8, respectively.

Laboratory tests showed that the erythrocyte sedimentation rate was 56 mm/h, and serum blood cell counts and C reactive protein levels were within normal range. Lumbar puncture showed an increased opening pressure, and cerebrospinal fluid (CSF) analysis revealed elevated white blood cell (WBC) count and protein levels (Table 1). Based on these findings, the initial clinical diagnosis of possible viral meningoencephalitis was achieved. The patient was treated empirically with intravenous acyclovir 10 mg/kg three times per day. However, his health status continued to gradually worsen, with the highest body temperature reaching 39°C, with confusion, occasional nonsense, and partial third cranial nerve palsy, on day 3 (Figure 1). The CASE and mRS scores were 13 and 4. Furthermore, CSF and serum analyses revealed the presence of anti-NMDAR antibody IgG, determined by cell-based assay (CBA) and tissue-based assay (TBA). The result of tuberculin-purified protein derivative (PPD) skin test was positive (++), and that of the T-SPOT.TB assay was positive. CSF was positive for the MTB complex DNA assay; however, the modified acid-fast staining and metagenomic next-generation sequencing (mNGS) result was negative. Moreover, MRI showed nodules and ring enhancement in the right lateral ventricle, left temporal and left cerebellar hemisphere, abnormal signal foci were observed in the right temporal lobe and basal ganglia, as well as enhancement of the pia mater of the right frontotemporal area (Figures 2C,D). Furthermore, the patient was negative for anti-MBP, anti-AQP4, anti-MOG, anti-GFAP, and anti-GQ1b IgGs, and neuron antibodies in serum and CSF. Tumor markers, blood cultures, and autoantibodies associated with other autoimmune diseases were also negative. Moreover, color Doppler ultrasound of lymphatic, reproductive, urinary, and digestive systems, and of the heart were all normal. The lungs were also normal as per bronchoscopy analysis.

**Table 1:** Some data of laboratory tests.

**Items**	**2021-8-5**	**2021-8-13**	**2021-8-18**	**2021-9-28**
Opening pressure (mm H_2_O)	325	280	170	140
CSF WBC (10^6^/ mm^3^)	12	480	80	34
CSF Protein (mg/dL)	1,650	1,000	720	540
CSF Glucose (mg/dL)	43.02	19.98	37.98	68.76
CSF Chlorine (mmol/L)	115	116.7	124.7	127.1
Blood glucose (mg/dL)	90	107.82	93.6	172.98
CSF Modified acid-fast staining	(–)	(–)	(±)^*^	(–)
CSF mNGS	(–)	–	–	–
CSF MTB complex DNA assay	–	(+)	(–)	(–)
CSF NMDAR-ab	1:3.2	–	–	1:3.2
Serum NMDAR-ab	1:1,000	–	–	1:32

* *Found one MTB;–, none tested*.

Diagnosis of tuberculosis meningoencephalitis combined with anti-NMDAR encephalitis was achieved, and a standard antituberculosis regimen (rifampicin 0.45 g/day, ethambutol 0.75 g/day, isoniazid 0.6 g/day, and pyrazinamide 1.5 g/day) and intravenous 1,000 mg/d glucocorticoid pulse therapy (halved every 3 days, and switched to oral after 12 days) was initiated. Thereafter, the consciousness status of the patient and irritability gradually improved, and the head and neck pain was substantially ameliorated. On day 8, despite the health improvements noticed, the comprehension, calculation ability, spatial learning, and memory impairments remained unchanged, and depression manifestations appeared. The pain symptoms disappeared, but neck resistance increased to 15°C. On day 13, a lumbar puncture was performed with intrathecal isoniazid (0.1 g) and dexamethasone (5 mg). CSF analysis revealed evident improvements (Table 1), and the comprehension, mood, verbal reduction, and partial oculomotor nerve palsy showed some improvements. The neck flexion angle of 25° remained. The CASE and mRS scores were 6 and 3. The patient was discharged with an oral standard antituberculosis regimen combined with prednisone (60 mg/day) for 2 weeks, which was tapered to 50 mg/day until the following medical examination.

After 1 month of follow-up, the patient had gained 8 kg without any discomfort. Physical examination showed only right ptosis and diplopia. Moreover, the CASE, MMSE, MoCA, and mRS scores were 3, 29, 27, and 1, respectively. The CSF anti-NMDAR antibody titer remained unchanged. Noteworthily, the serum anti-NMDAR antibody titer, and protein and WBC of CSF were significantly reduced (Table 1). Brain MRI revealed significant shrinkage of the primary nodules. Nevertheless, the lesion in the right basal ganglia was larger than before, new lesions appeared in the right pontine and right-sided temporal lobe, and the right frontotemporal leptomeninges showed more evident enhancement than before (Figures 2E,F). The patient continued to receive the antituberculosis treatment regimen along with regular tapered prednisone. Additional evaluations revealed that the patient was still experiencing mild visual acuity decline in the right eye but without impact on his daily life, and the ptosis and memory disorders disappeared without further complaints. Physical examination remarkably improved after 3 months of discharge. The CASE and mRS scores were 2 and 1. The lesions on the CT chest and brain MRI (Figure 2G) showed evident improvements.

## Discussion and Conclusion

Although no direct evidence for tuberculosis-induced anti-NMDAR encephalitis, our patient was diagnosed with tuberculosis meningoencephalitis associated with anti-NMDAR encephalitis (5). According to the clinical symptoms, basic laboratory data, thickened meninges and granuloma in MRI, and MTB DNA detection in the CSF, the patient met the criteria for tuberculous meningoencephalitis and pleurisy diagnosis (6). However, it remains unclear why the mNGS failed to identify the MTB. It is possible that small populations of non-replicating MTB may persist in granulomatous foci (7), almost few MTB may be contained in CSF and serum. In addition, mNGS has limited sensitivity for the detection of intracellular bacteria; indeed, a meta-analysis of mNGS for MTB diagnosis revealed a comprehensive sensitivity was 61% (8). Notably, at early disease onset, irritability, cognitive dysfunction (comprehension, orientation, and calculation), and verbal reduction observed in our patient could not be explained by intracranial lesions on MRI. Furthermore, TBA and CBA methods positively detected anti-NMDAR antibodies in the blood and CSF, whereas neoplastic disorders, viral encephalitis, rheumatologic disorders, bacterial endocarditis, and other disease were excluded (5). Importantly, as tuberculous meningoencephalitis could not be ruled out, possible anti-NMDAR encephalitis was diagnosed.

In various animal models of tuberculosis, bacterial growth is rapid during the first 3 weeks of infection and reaches a plateau when adaptive immunity develops (9, 10). In addition, tuberculous granuloma formation requires adaptive immunity (11). Our patient experienced headaches only in the first 20 days; thus, we believe that the MTB infection occurred before the anti-NMDAR encephalitis was developed.

As no such case had been previously reported, and the etiology and pathogenesis of anti-NMDAR encephalitis are not fully understood, we questioned the probability of other causes of anti-NMDAR encephalitis. First reports on anti-NMDAR encephalitis described a close relationship with tumors (2). However, in this case, the patient was negative for serum tumor markers and CSF anti-tumor-associated neuron antibodies, and symptoms or imaging data of lymphatic, hematopoietic, reproductive, urinary, digestive, or respiratory cancers were not observed. Moreover, tumor-related manifestations were noted during the follow-up period. Interestingly, some cases of non-tumor-associated anti-NMDAR encephalitis were described to be related to infections (12, 13), in particular, HSV infection (14). Our patient presented with headache, fever, and signs of meningeal irritation, which strongly suggested CNS infections. Viral meningoencephalitis was the first diagnosis; however, 26 days after disease onset, the symptoms and signs were further exacerbated, and the antiviral therapy was ineffective. Moreover, CSF and brain MRI data failed to support this initial diagnosis. To date, anti-NMDAR encephalitis has been rarely associated with multiple sclerosis (15) and vaccines (16, 17); nevertheless, our patient was negative for anti-MBP, anti-AQP4, anti-MOG, and anti-GFAP IgGs in the serum, and CSF, or had clinical signs or symptoms of such etiology. Moreover, the patient had not been vaccinated in 10 years. Therefore, common autoimmune demyelinating diseases of the CNS or vaccine-triggered pathology were excluded from the differential diagnosis.

The mechanisms connecting anti-NMDAR encephalitis with MTB infection remain unclear. Although we could not obtain a direct proof of the relation between these two conditions, there are some possible explanations. Tuberculosis infection itself triggers immune responses, which in turn may establish a potential background that favors autoimmunity. In the case of our patient, the MTB infection may have led to anti-NMDAR encephalitis development.

The MTB bacteria are phagocytosed by macrophages, which in turn produce large amounts of signaling molecules (such as TNF-α, IL-6, and IL-12p40) that recruit other immune cells (18). Moreover, lipids of the bacterial cell wall can destroy the mitochondrial membrane, which affects the cellular energy metabolism, while bacterial lipids and proteins can induce delayed hypersensitivity, leading to tissue necrosis and systemic symptoms, activation of the CD4^+^/CD8^+^ T cells, and enhanced secretion of cytokines (11). These strong immunities and inflammatory responses can derange the intracranial immune microenvironment, directly damaging the nerve tissue and promot leakage of NMDAR antigens; they can impair the blood-brain-barrier, increasing its permeability and allowing for the peripheral analogs of the NMDAR antigen to enter the intracranial space; then trigger the production of anti-NMDAR antibodies (19). Nevertheless, other unknown immunological triggers may have been involved in this case, and the tuberculosis infection may have appeared coincidently with anti-NMDAR encephalitis.

Anti-NMDAR encephalitis patients usually present with abnormal behavior and mental, speech dysfunction, dyskinesias, memory deficits, autonomic instability, and a decrease in the level of consciousness (5). Neither oculomotor nerve palsy nor neck resistance has been associated with anti-NMDAR encephalitis. Moreover, non-specific lesions on MRI were identified in 33–50% of anti-NMDAR encephalitis patients. To date, no intracranial granuloma was reported in an anti-NMDAR encephalitis patient, and only one case report described microglial nodules in the frontal lobes and basal ganglia (20). Nonetheless, oculomotor nerve palsy, neck resistance, and intracranial granuloma on MRI are characteristic features of MBT meningoencephalitis (14). As Bickerstaff's brainstem encephalitis (BBE) is characterized by abnormal mental status, bilateral external ophthalmoplegia and ataxia, and presence of anti-GQ1b antibodies, this disorder could have also been considered as differential diagnosis in our case. Despite 32% of patients do not have detectable antibodies (21), our patient had unilateral external ophthalmoplegia and not ataxia, so he did not meet the BBE criteria.

Tuberculosis combined with anti-NMDAR encephalitis is a rare event; nonetheless, it should be considered when patients with tuberculosis present with atypical symptoms. Furthermore, tuberculosis should be considered when patients with anti-NMDAR encephalitis present considerable weight loss pre-onset and/or brain granulomas; various laboratory methods should be employed to detect tuberculosis and prevent its spread after using immunosuppressants. Follow-up and restaging MRI assessments are a good strategy to diagnose such cases and evaluate treatment efficacy. In summary, the present case suggests that tuberculosis may trigger or aggravate anti-NMDAR encephalitis, an association that should be recognized to avoid misdiagnosis and ensure adequate patient care.

## Data Availability Statement

The original contributions presented in the study are included in the article/supplementary material, further inquiries can be directed to the corresponding authors.

## Ethics Statement

Written informed consent was obtained from the individual(s) for the publication of any potentially identifiable images or data included in this article.

## Author Contributions

CX: data acquisition and drafting the manuscript. WQ: data acquisition. LJ and YH: revision of the manuscript. CS: data interpretation and critical revision of the manuscript. All authors read and approved the final version of the manuscript.

## Funding

This study was supported by a grant from the National Natural Science Foundation of China (grant number: 33020105348).

## Conflict of Interest

The authors declare that the research was conducted in the absence of any commercial or financial relationships that could be construed as a potential conflict of interest.

## Publisher's Note

All claims expressed in this article are solely those of the authors and do not necessarily represent those of their affiliated organizations, or those of the publisher, the editors and the reviewers. Any product that may be evaluated in this article, or claim that may be made by its manufacturer, is not guaranteed or endorsed by the publisher.
